# Supplementary material for: Leptin antagonism improves Rett syndrome phenotype in symptomatic Mecp2-deficient mice
Source: Neurotherapeutics. 2026 Apr 18;23(3):e00910. doi: 10.1016/j.neurot.2026.e00910 (PMC13098445; doi:10.1016/j.neurot.2026.e00910)
Supplement: Multimedia component 1 [file mmc1.docx]

**SUPPLEMENTARY FIGURES**


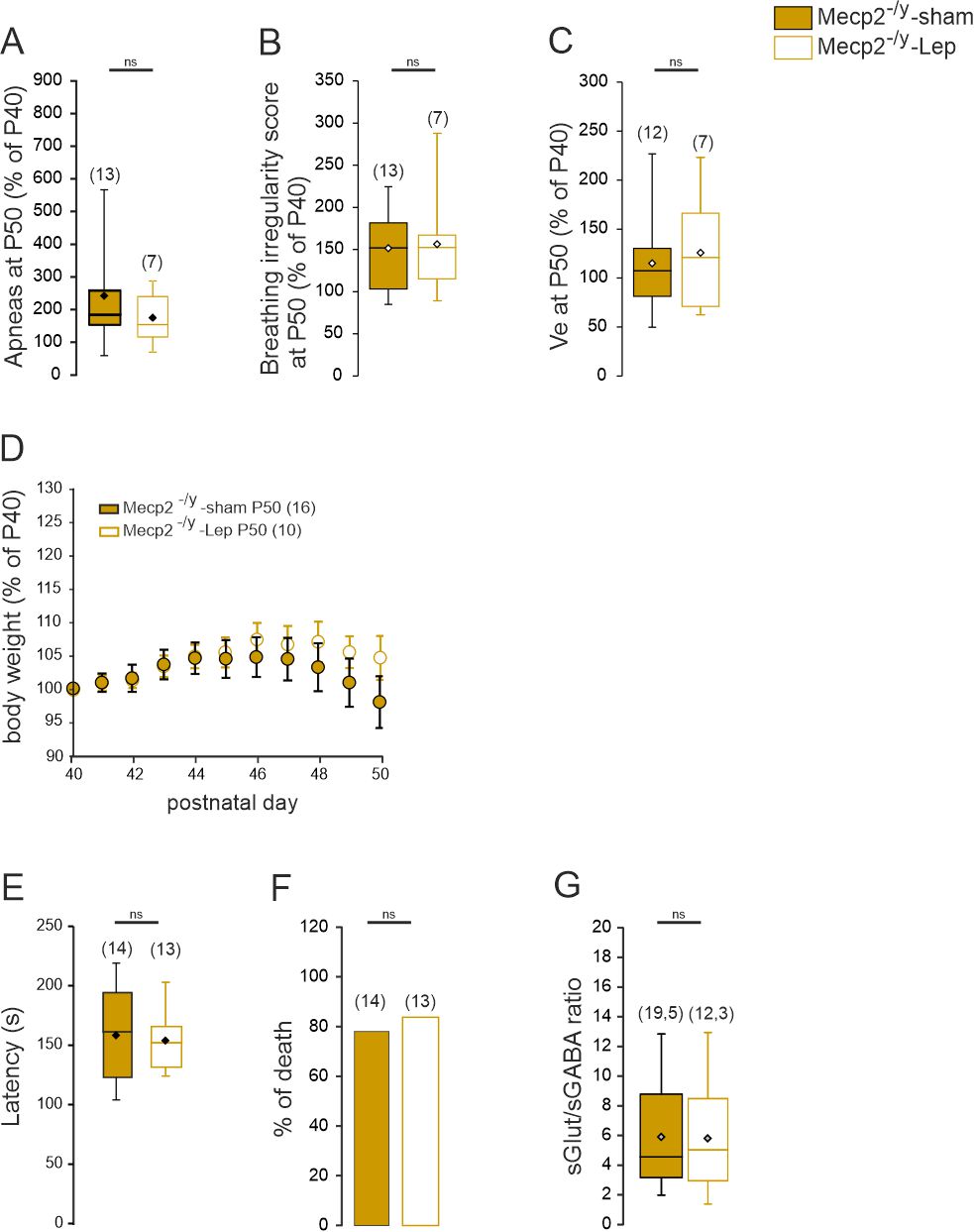


**Supplementary Fig S1: Leptin treatment has no effect on *Mecp2^-/y^* mice.**

P40 *Mecp2^-/y^* received daily sub-cutaneous injection of leptin recombinant (5µg/g) during 10 days. Sham mice received the same volume of vehicle. **A-C**) Box plots of the percentage of change (% of P40) of apnea frequency (**A**), breathing irregularity score (**B**) and minute ventilation (**C**) in sham- and treated- *Mecp2*^-/y^ mice. **D**) Body weight change (% of P40) as a function of age in sham- anti-leptin treated *Mecp2^-/y^* mice. **E)** Box plots of the frequency ratio of spontaneous glutamatergic and GABAergic postsynaptic currents recorded on CA3 pyramidal neurons of sham- and leptin treated- *Mecp2*^-/y^ mice at P50. Numbers in parenthesis indicate the number of mice used. Two-tailed unpaired t-test.


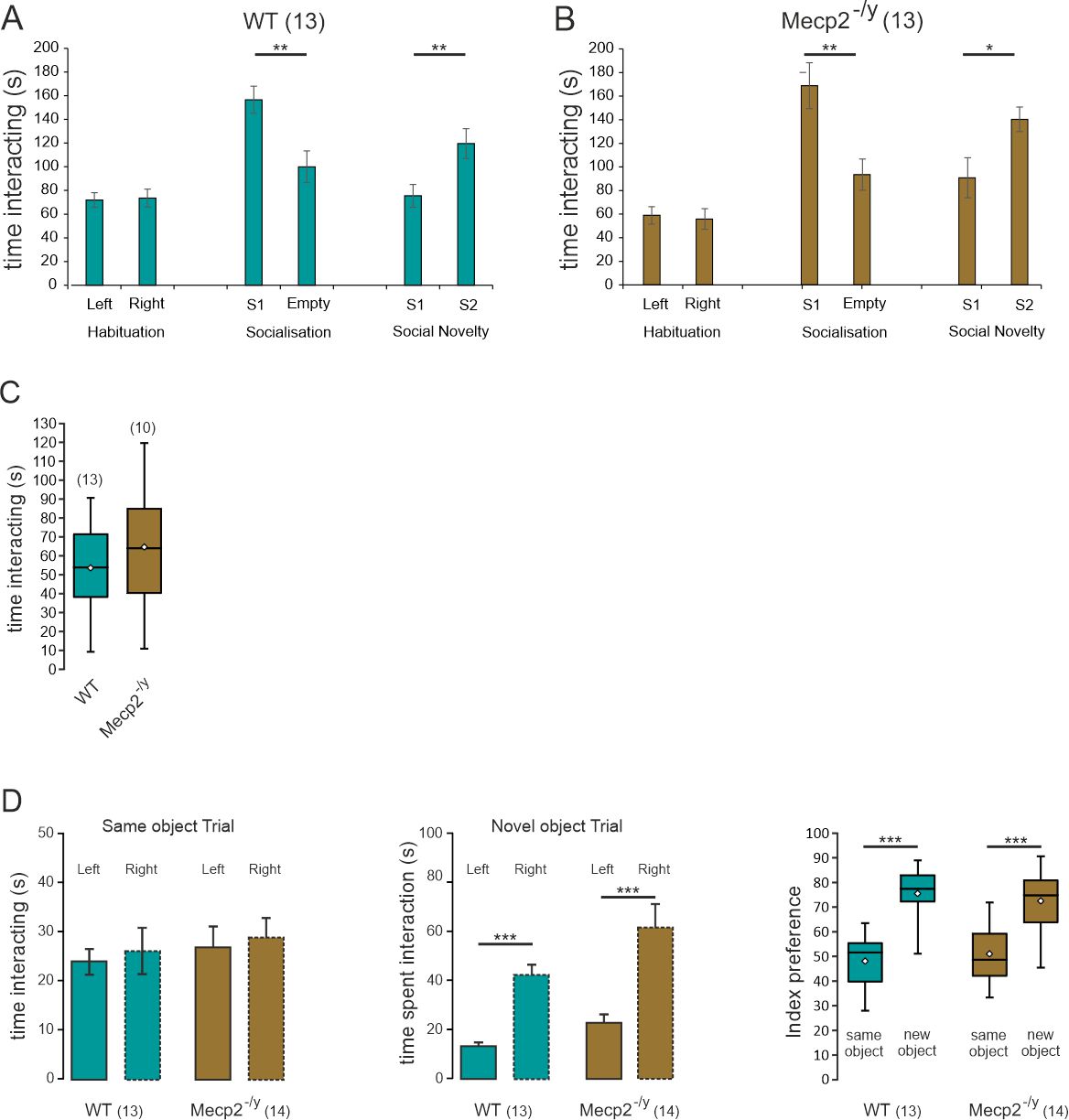


**Supplementary Fig S2: Lack of differences in social and cognitive behavior between P50 WT and *Mecp2^-/y^* mice.**

**A, B**) Mean + SEM plots of the time interacting of P50 WT (A) and *Mecp2^-/y^* (B) mice during the different phase of the 3 chambers test. **C**) Box plots of the time interacting of P50 WT and *Mecp2^-/y^* mice with a stranger mouse in the spontaneous social interaction test. **D**) Mean + SEM plots of the time interacting and index preference of P50 WT and *Mecp2^-/y^* mice in the novel object recognition test. Numbers in parenthesis indicate the number of mice used. ***P* < 0.01; ****P* < 0.001, two-tailed paired *t*-test (A, B, D), two-tailed unpaired *t*-test (B, D).


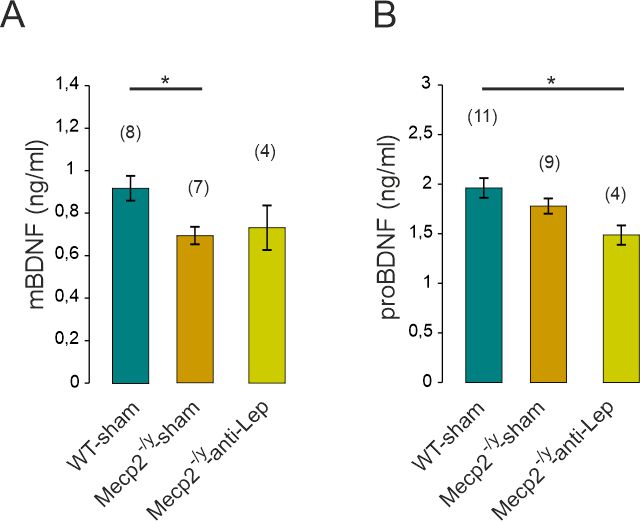


**Supplementary Fig S3: mature and pro-brain derived neurotrophic factor (BDNF) proteins expression in WT and *Mecp2^-/y^* mice.**

**A-C**) Box plots of mature (A) and pro (B) BDNF protein expressions in hippocampal samples taken from P50 WT and *Mecp2^-/y^* mice, using ELISA kit. P40 WT and *Mecp2^-/y^* received sub-cutaneous injection of vehicle (sham) or anti-leptin (5µg/g), every other day, during 10 days. **P* < 0.05, One-way ANOVA followed by a Tukey’s multiple comparison.

**STATISTICAL TABLES**

**Table S1: Statistical differences among the samples illustrated in Fig. 1.** Peach highlighting indicates cases when the difference is statistically significant at 0.05 level (*P*<0.05).

| Data Reference | Distribution | Type of test | Power | n |
| --- | --- | --- | --- | --- |
| **Fig. 1A**  WT age dependence  Mecp2-/y age dependence  P5 vs P15  P5 vs P50  P30 vs P50 | Normal | One-way ANOVA  One-way ANOVA  Post-hoc tukey  Post-hoc tukey  Post-hoc tukey | *F*(2,40)=1.04, p=0.8  *F*(2,29)=4.27, p=0.02  p=0.99  p=0.003  p=0.09 | 12, 10, 21  11, 6, 15  11, 6  6, 15  11, 15 |
| **Fig. 1C**  Visceral WT vs Mepc2-/y  Inguinal WT vs Mepc2-/y | Normal | Unpaired *t*-test  Unpaired *t*-test | t=4.56, df=19, p=0.0002  t=0.41, df=16, p=0.68 | 9, 12  8, 10 |
| **Fig. 1D**  P30 WT vs Mepc2-/y  P50 WT vs Mepc2-/y | Normal | Unpaired *t*-test  Unpaired *t*-test | t=1.75, df=22, p=0.097  t=3.67, df=13, p=0.0028 | 11, 13  7, 8 |
| **Fig. 1E**  P30 WT vs Mepc2-/y  P50 WT vs Mepc2-/y | Normal | Unpaired *t*-test  Unpaired *t*-test | t=2.24, df=6, p=0.066  t=2.65, df=11, p=0.026 | 4, 4  5, 9 |
| **Fig. F**  P30 WT vs Mepc2-/y  P50 WT vs Mepc2-/y | Normal | Unpaired *t*-test  Unpaired *t*-test | t=2.36, df=17, p=0.03  t=2.43, df=10, p=0.035 | 10, 9  6,6 |
| **Fig. 1G**  **LH**  WT sham, WT lep, Mepc2-/y sham  WT sham vs WT lep  WT sham vs Mepc2-/y sham  Mepc2-/y sham vs WT lep  Mepc2-/y sham vs Mepc2-/y lep, Mepc2-/y anti-lep  Mepc2-/y sham vs Mepc2-/y lep  Mepc2-/y sham vs Mepc2-/y anti-lep  **DM**  WT sham, WT lep, Mepc2-/y sham  WT sham vs WT lep  WT sham vs Mepc2-/y sham  Mepc2-/y sham vs WT lep  Mepc2-/y sham vs Mepc2-/y lep, Mepc2-/y anti-lep  Mepc2-/y sham vs Mepc2-/y lep  Mepc2-/y sham vs Mepc2-/y anti-lep  **VMH**  WT sham, WT lep, Mepc2-/y sham  WT sham vs WT lep  WT sham vs Mepc2-/y sham  Mepc2-/y sham vs WT lep  Mepc2-/y sham vs Mepc2-/y lep, Mepc2-/y anti-lep  Mepc2-/y sham vs Mepc2-/y lep  Mepc2-/y sham vs Mepc2-/y anti-lep  **Arc**  WT sham, WT lep, Mepc2-/y sham  WT sham vs WT lep  WT sham vs Mepc2-/y sham  Mepc2-/y sham vs WT lep  Mepc2-/y sham vs Mepc2-/y lep, Mepc2-/y anti-lep  Mepc2-/y sham vs Mepc2-/y lep  Mepc2-/y sham vs Mepc2-/y anti-lep  **PVN**  WT sham, WT lep, Mepc2-/y sham  WT sham vs WT lep  WT sham vs Mepc2-/y sham  Mepc2-/y sham vs WT lep  Mepc2-/y sham vs Mepc2-/y lep, Mepc2-/y anti-lep  Mepc2-/y sham vs Mepc2-/y lep  Mepc2-/y sham vs Mepc2-/y anti-lep | Normal | One-way ANOVA  Post-hoc tukey  Post-hoc tukey  Post-hoc tukey  One-way ANOVA  Post-hoc Dunnet  Post-hoc Dunnet  One-way ANOVA  Post-hoc tukey  Post-hoc tukey  Post-hoc tukey  One-way ANOVA  Post-hoc Dunnet  Post-hoc Dunnet  One-way ANOVA  Post-hoc tukey  Post-hoc tukey  Post-hoc tukey  One-way ANOVA  Post-hoc Dunnet  Post-hoc Dunnet  One-way ANOVA  Post-hoc tukey  Post-hoc tukey  Post-hoc tukey  One-way ANOVA  Post-hoc Dunnet  Post-hoc Dunnet  One-way ANOVA  Post-hoc tukey  Post-hoc tukey  Post-hoc tukey  One-way ANOVA  Post-hoc Dunnet  Post-hoc Dunnet | F(2,12)=0.97, p<0.0001  p<0.0001  p<0.0001  p=0.97  F(2,11)=8.05, p=0.007  p=0.089  P=0.089  F(2,12)=1.3, p<0.0001  p<0.0001  p=0.0002  p=0.06  F(2,11)=2.18, p=0.16  p=0.17  p=0.26  F(2,12)=0.49, p<0.0001  p<0.0001  p=0.0002  p=0.83  F(2,11)=24.7, p<0.0001  p=0.004  p=0.015  F(2,12)=0.86, p=0.0003  p<0.002  p=0.0004  p=0.56  F(2,11)=7.9, p<0.007  p=0.01  p=0.96  F(2,12)=4.37, p=0.0003  p<0.0003  p<0.0001  p=0.35  F(2,11)=1.9, p=0.17  p=0.5  p=0.12 | 5, 5, 5  5, 5  5, 5  5, 5  5,5,4  5,5  5,4  5, 5, 5  5, 5  5, 5  5, 5  5.5.4  5.5  5.4  5, 5, 5  5, 5  5, 5  5, 5  5.5.4  5.5  5.4  5, 5, 5  5, 5  5, 5  5, 5  5.5.4  5.5  5.4  5, 5, 5  5, 5  5, 5  5, 5  5.5.4  5.5  5.4 |

**Table S2: Statistical differences among the samples illustrated in Fig. 2.** Peach highlighting indicates cases when the difference is statistically significant at 0.05 level (*P*<0.05).

| Data Reference | Distribution | Type of test | Power | n |
| --- | --- | --- | --- | --- |
| **Fig. 2A** | Normal | Unpaired *t*-test | t=8.12, df=55, p<0.0001 | 25, 26 |
| **Fig. 2B**  WT sham P40 vs P50  WT Lep P40 vs P50 | Normal | paired *t*-test  paired *t*-test | t=0.59, df=18, p=0.56  t=2.37, df=28, p=0.02 | 8  15 |
| **Fig. 2C**  Mepc2-/y sham P40 vs P50  Mepc2-/y antilep P40 vs P50 | Normal | paired *t*-test  paired *t*-test | t=4.6, df=27, p<0.0001  t=0.69, df=18, p=0.49 | 13  11 |
| **Fig. 2D** | Non-normal | Mann Whitney U test | U=148, p=0.0007 | 25,26 |
| **Fig. 2E**  WT sham vs WT lep  WT sham, Mepc2-/y sham, Mepc2-/y antilep  WT sham vs Mepc2-/y sham  WT sham vs Mepc2-/y antilep  Mepc2-/y sham vs Mepc2-/y antilep | Normal | Unpaired *t*-test  One-way ANOVA  Post-hoc tukey  Post-hoc tukey | t=2.17, df=23, p=0.04  F(2,28)=2.85, p=0.0001  p=0.015  p=0.75  p=0.004 | 8, 15  8, 13, 10  8, 13  8, 15  13, 15 |
| **Fig. 2F** | Non-normal | Mann Whitney U test | U=75, p<0.0001 | 25, 26 |
| **Fig. 2G**  WT sham vs WT lep  WT sham, Mepc2-/y sham, Mepc2-/y antilep  WT sham vs Mepc2-/y sham  WT sham vs Mepc2-/y antilep  Mepc2-/y sham vs Mepc2-/y antilep | Normal | Unpaired *t*-test  One-way ANOVA  Post-hoc tukey  Post-hoc tukey  Post-hoc tukey | t=0.35, df=19, p=0.72  F(2,25)=1.34, p=0.018  p=0.15  p=0.56  p=0.62 | 8, 15  8, 13, 10  8, 13  8, 15  13, 15 |

**Table S3: Statistical differences among the samples illustrated in Fig. 3.** Peach highlighting indicates cases when the difference is statistically significant at 0.05 level (*P*<0.05).

| Data Reference | Distribution | Type of test | Power | n |
| --- | --- | --- | --- | --- |
| **Fig. 3A** | Normal | Unpaired *t*-test | t=10.8,df=37, p<0.0001 | 12, 27 |
| **Fig. 3C**  WT sham vs WT lep  WT sham, Mepc2-/y sham, Mepc2-/y antilep  WT sham vs Mepc2-/y sham  WT sham vs Mepc2-/y antilep  Mepc2-/y sham vs Mepc2-/y antilep | Normal | Unpaired *t*-test  One-way ANOVA  Post-hoc tukey  Post-hoc tukey  Post-hoc tukey | t=4.31, df=62, p<0.0001  F(2,77)=12.57,p<0.0001  p=0.001  p=0.045  p<0.0001 | 43, 21  43, 19, 18  43, 19  43, 18  19, 18 |
| **Fig. 3D** | Normal | Unpaired *t*-test | t=4.13, df=37, p=0.002 | 12, 27 |
| **Fig. 3E**  Posture  General aspect  Spontaneous activity  Limb grasp  Tremor | Non-normal  Non-normal  Normal  Non-Normal  Non-normal | Mann Whitney U test  Mann Whitney U test  Unpaired *t*-test  Mann Whitney U test Mann Whitney U test | U=132, p=0.16  U=108, p=0.03  t=5.66, df=37, p<0.0001  U=102, p=0.022  U=96, p=0.016 | 12, 27  12, 27  12, 27  12, 27  12, 27 |
| **Fig. 3F** |  | Kaplan- Meier log rank test | P=0.16 | 18, 14 |
| **Fig. 3G**  Mepc2-/y sham: P40 vs P50  Posture  General aspect  Spontaneous activity  Limb grasp  Tremor  Mepc2-/y antilep: P40 vs P50  Posture  General aspect  Spontaneous activity  Limb grasp  Tremor | Non-normal  Non-normal  Normal  Non-Normal  Normal  Non-normal  Non-normal  Normal  Non-Normal  Normal | Wilcoxon matched-pairs signed rank test  Wilcoxon matched-pairs signed rank test  Paired *t*-test  Wilcoxon matched-pairs signed rank test  Paired *t*-test  Wilcoxon matched-pairs signed rank test  Wilcoxon matched-pairs signed rank test  Paired *t*-test  Wilcoxon matched-pairs signed rank test  Paired *t*-test | W=-55, p=0.06  W=-33, p=0.03  t=4.66, df=12, p=0.0005  W=-36, p=0.007  t=3.5, df=12, p=0.003  W=0, p>0.999  W=-9, p=0.25  t=3.8, df=12, p=0.002  W=-46, p=0.015  t=0.89, df=12, p=0.39 | 13  13 |

**Table S4: Statistical differences among the samples illustrated in Fig. 4.** Peach highlighting indicates cases when the difference is statistically significant at 0.05 level (*P*<0.05).

| Data Reference | Distribution | Type of test | Power | n |
| --- | --- | --- | --- | --- |
| **Fig. 4A** | Normal | Unpaired *t*-test | t=2.55, df=27, p=0.016 | 14, 15 |
| **Fig. 4B**  Mepc2-/y sham: P40 vs P50  Mepc2-/y antilep: P40 vs P50 | Normal  Normal | Paired *t*-test  Paired *t*-test | t=2.96, df=7, p=0.03  t=0.18, df=7, p=0.85 | 8  8 |
| **Fig. 4C**  WT sham vs WT lep  WT sham, Mepc2-/y sham, Mepc2-/y antilep  WT sham vs Mepc2-/y sham  WT sham vs Mepc2-/y antilep  Mepc2-/y sham vs Mepc2-/y antilep | Normal  Normal | Unpaired *t*-test  One-way ANOVA  Post-hoc tukey  Post-hoc tukey  Post-hoc tukey | t=1.09, df=15, p=0.29  F(2,22)=0.49, p=0.02  p=0.03  p>0.999  p=0.049 | 8, 8  8, 8, 8 |
| **Fig. 4D** | Non-normal | Mann Whitney U test | U=29, p<0.0001 | 18, 20 |
| **Fig. 4E**  Mepc2-/y sham: P40 vs P50  Mepc2-/y antilep: P40 vs P50 | Normal  Normal | Paired *t*-test  Paired *t*-test | t=1.13, df=17, p=0.27  t=0.33, df=34, p=0.74 | 18  18 |
| **Fig. 4F**  WT sham vs WT lep  WT sham, Mepc2-/y sham, Mepc2-/y antilep  WT sham vs Mepc2-/y sham  WT sham vs Mepc2-/y antilep  Mepc2-/y sham vs Mepc2-/y antilep | Normal  Normal | Unpaired *t*-test  One-way ANOVA  Post-hoc tukey  Post-hoc tukey  Post-hoc tukey | t=1.06, df=15, p=0.3  F(2, 41)=6.6, p<0.0001  p<0.0001  p=0.0008  p=0.52 | 8, 9  8, 18, 18  8, 18  18, 18  8, 18 |
| **Fig. 4G**  WT vs Mepc2-/y  WT sham vs WT lep  WT sham, Mepc2-/y sham, Mepc2-/y antilep  WT sham vs Mepc2-/y sham  WT sham vs Mepc2-/y antilep  Mepc2-/y sham vs Mepc2-/y antilep | Normal  Normal  Normal | Unpaired *t*-test  Unpaired *t*-test  One-way ANOVA  Post-hoc tukey  Post-hoc tukey  Post-hoc tukey | t=9.10, df=24, p<0.0001  t=1.03, df=15, p=0.21  F(2, 21)=3.6, p<0.001  P=0.0002  P<0.0001  P=0.52 | 13, 13  8, 9  8, 7, 9  8, 7  8, 9  7, 9 |
| **Fig. 4H**  WT vs Mepc2-/y  WT sham vs WT lep  WT sham, Mepc2-/y sham, Mepc2-/y antilep  WT sham vs Mepc2-/y sham  WT sham vs Mepc2-/y antilep  Mepc2-/y sham vs Mepc2-/y antilep | Normal  Normal  Normal | Unpaired *t*-test  Unpaired *t*-test  One-way ANOVA  Post-hoc tukey  Post-hoc tukey  Post-hoc tukey | t=2.86, df=24, p=0.008  t=0.97, df=15, p=0.34  F(2, 21) =4.09, p=0.03  p=0.08  p=0.003  p=0.42 | 13, 13  8, 9  8, 7, 9  8, 7  8, 9  7, 9 |

**Table S5: Statistical differences among the samples illustrated in Fig. 5.** Peach highlighting indicates cases when the difference is statistically significant at 0.05 level (*P*<0.05).

| Data Reference | Distribution | Type of test | Power | n |
| --- | --- | --- | --- | --- |
| **Fig. 5B**  WT vs Mepc2-/y  WT sham vs WT lep  WT sham, Mepc2-/y sham, Mepc2-/y antilep  WT sham vs Mepc2-/y sham  WT sham vs Mepc2-/y antilep  Mepc2-/y sham vs Mepc2-/y antilep | Normal  Normal  Normal | Unpaired *t*-test  Unpaired *t*-test  One-way ANOVA  Post-hoc tukey  Post-hoc tukey  Post-hoc tukey | t=3.8, df=25, p=0.0008  t=4.51, df=34, p<0.0001  F(2,48)=11.5, p<0.0001  p<0.0001  p=0.0003  p=0.99 | 12, 15  20, 16  20, 19, 16  20, 19  20, 16  19, 16 |
| **Fig. 5C**  WT vs Mepc2-/y  WT sham, Mepc2-/y sham, Mepc2-/y antilep  WT sham vs Mepc2-/y sham  WT sham vs Mepc2-/y antilep  Mepc2-/y sham vs Mepc2-/y antilep | Non-normal  Non-normal | Mann Whitney U test  Kruskal-Wallis test  Post-hoc dunn’s  Post-hoc dunn’s  Post-hoc dunn’s | U=12, p=0.015  X^2^=10.7, p<0.0048  P=0.052  p>0.999  p=0.006 | 11, 7  11, 19, 21  11, 19  11, 21  19, 21 |
| **Fig. 5D**  Early potentiation  Late potentiation | Normal  Normal | Two-way RM ANOVA  Two-way RM ANOVA | F(1,22)=6.7, p=0.016  F(1,16)=5.1, p=0.03 | 11, 7 |
| **Fig. 5E**  Early potentiation  Late potentiation | Normal  Normal | Two-way RM ANOVA  Two-way RM ANOVA | F(1,22)=11.9, p=0.2  F(1,20)=2.1, p=0.16 | 9, 11  9, 11 |
| **Fig. 5F**  Early potentiation  Late potentiation | Normal  Normal | Two-way RM ANOVA  Two-way RM ANOVA | F(1,14)=4.59, p=0.05  F(1,11)=3.7, p=0.07 | 5, 8  5, 8 |

**Table S6: Statistical differences among the samples illustrated in Fig. 6.** Peach highlighting indicates cases when the difference is statistically significant at 0.05 level (*P*<0.05).

| Data Reference | Distribution | Type of test | Power | n |
| --- | --- | --- | --- | --- |
| **Fig. 6A**  WT, Mecp2-/y, Mecp2y/-;ob/+  WT vs Mecp2-/y  WT vs Mecp2y/-;ob/+  Mecp2-/y vs Mecp2y/-;ob/+ | Normal | One-way ANOVA  Post-hoc tukey  Post-hoc tukey  Post-hoc tukey | F(2,16)=3.05, p=0.007  p=0.007  p=0.66  p=0.048 | 6, 7, 6  6, 7  6, 6  7, 6 |
| **Fig. 6B**  WT, Mecp2-/y, Mecp2y/-;ob/+ at P40  WT vs Mecp2-/y  WT vs Mecp2y/-;ob/+  Mecp2-/y vs Mecp2y/-;ob/+  WT, Mecp2-/y, Mecp2y/-;ob/+ at P50  WT vs Mecp2-/y  WT vs Mecp2y/-;ob/+  Mecp2-/y vs Mecp2y/-;ob/+ | Normal  Normal | One-way ANOVA  Post-hoc tukey  Post-hoc tukey  Post-hoc tukey  One-way ANOVA  Post-hoc tukey  Post-hoc tukey  Post-hoc tukey | F(2,20)=6.45, p=0.0006  p=0.005  p=0.042  p=0.144  F(2,17)=21.0, p<0.001  P<0.0001  p=0.013  p<0.001 | 9, 6, 8  9, 6  9, 8  6, 8  9, 4, 7  9, 4  9, 7  4, 7 |
| **Fig. 6C** |  | Mann Whitney U test | t=2.28, df=9, p=0.04 | 4, 7 |
| **Fig. 6D**  WT, Mecp2-/y, Mecp2y/-;ob/+ at P40  WT vs Mecp2-/y  WT vs Mecp2y/-;ob/+  Mecp2-/y vs Mecp2y/-;ob/+  WT, Mecp2-/y, Mecp2y/-;ob/+ at P50  WT vs Mecp2-/y  WT vs Mecp2y/-;ob/+  Mecp2-/y vs Mecp2y/-;ob/+ | Normal  Normal | One-way ANOVA  Post-hoc tukey  Post-hoc tukey  Post-hoc tukey  One-way ANOVA  Post-hoc tukey  Post-hoc tukey  Post-hoc tukey | F(2,48)=0.18, p<0.0001  p<0.0001  p<0.0001  p=0.02  F(2,43)=1.18, <0.0001  p<0.0001  p<0.0001  p=0.002 | 9, 19, 24  9, 19  9, 24  18, 24  11, 15, 20  11, 15  11, 20  15, 20 |
| **Fig. 4E**  WT, Mecp2-/y, Mecp2y/-;ob/+  WT vs Mecp2-/y  WT vs Mecp2y/-;ob/+  Mecp2-/y vs Mecp2y/-;ob/+ | Normal | One-way ANOVA  Post-hoc tukey  Post-hoc tukey  Post-hoc tukey | F(2,37)=12.7, p<0.0001  p<0.0001  p=0.39  p=0.0008 | 11, 12, 17  11, 12  11, 17  12, 17 |
| **Fig. 6F**  Early potentiation  Late potentiation | Normal  Normal | Two-way RM ANOVA  Two-way RM ANOVA | F(1,18)=2.41, p=0.13  F(1,13)=4.23, p=0.057 | 7,7  7, 7 |
| **Fig. 6G**  WT, Mecp2-/y, Mecp2y/-;ob/+ at P40  WT vs Mecp2-/y  WT vs Mecp2y/-;ob/+  Mecp2-/y vs Mecp2y/-;ob/+  WT, Mecp2-/y, Mecp2y/-;ob/+ at P50  WT vs Mecp2-/y  WT vs Mecp2y/-;ob/+  Mecp2-/y vs Mecp2y/-;ob/+ | Normal  Normal | One-way ANOVA  Post-hoc tukey  Post-hoc tukey  Post-hoc tukey  One-way ANOVA  Post-hoc tukey  Post-hoc tukey  Post-hoc tukey | F(2,31)=11.6, p=0.0015  p=0.001  p=0.06  p=0.1  F(2,18)=4.0, p=0.0003  p=0.0006  p=0.0007  p=0.999 | 8, 11, 15  8, 11  8, 15  11, 15  6, 9, 8  6, 9  6, 8  9, 8 |
| **Fig. 6H**  WT, Mecp2-/y, Mecp2y/-;ob/+ at P40  Posture  General aspect  Spontaneous activity  WT vs Mecp2-/y  WT vs Mecp2y/-;ob/+  Mecp2-/y vs Mecp2y/-;ob/+  Limb grasp  WT vs Mecp2-/y  WT vs Mecp2y/-;ob/+  Mecp2-/y vs Mecp2y/-;ob/+  Tremor  WT vs Mecp2-/y  WT vs Mecp2y/-;ob/+  Mecp2-/y vs Mecp2y/-;ob/+  WT, Mecp2-/y, Mecp2y/-;ob/+ at P50  General aspect  WT vs Mecp2-/y  WT vs Mecp2y/-;ob/+  Mecp2-/y vs Mecp2y/-;ob/+  Spontaneous activity  WT vs Mecp2-/y  WT vs Mecp2y/-;ob/+  Mecp2-/y vs Mecp2y/-;ob/+  Limb grasp  WT vs Mecp2-/y  WT vs Mecp2y/-;ob/+  Mecp2-/y vs Mecp2y/-;ob/+  Tremor  WT vs Mecp2-/y  WT vs Mecp2y/-;ob/+  Mecp2-/y vs Mecp2y/-;ob/+ | Non-normal  Non-normal  Normal  Non-normal  Non-normal  Non-Normal  Normal  Non-normal  Non-normal | Kruskal-Wallis test  Kruskal-Wallis test  One-way ANOVA  Post-hoc tukey  Post-hoc tukey  Post-hoc tukey  Kruskal-Wallis test  Post-hoc Dunn’s  Post-hoc Dunn’s  Post-hoc Dunn’s  Kruskal-Wallis test  Post-hoc Dunn’s  Post-hoc Dunn’s  Post-hoc Dunn’s  Kruskal-Wallis test  Post-hoc Dunn’s  Post-hoc Dunn’s  Post-hoc Dunn’s  One-way ANOVA  Post-hoc tukey  Post-hoc tukey  Post-hoc tukey  Kruskal-Wallis test  Post-hoc Dunn’s  Post-hoc Dunn’s  Post-hoc Dunn’s  Kruskal-Wallis test  Post-hoc Dunn’s  Post-hoc Dunn’s  Post-hoc Dunn’s | X^2^=2.09, p=0.35  X^2^=2.6, p=0.26  F(2,31)=7.6, p=0.0001  p<0.0001  p=0.0048  p=0.14  X^2^=7.1, p=0.028  p=0.03  p=0.9  p=0.17  X^2^=7.8, p=0.016  p=0.019  p=0.08  p>0.999  X^2^=9.1, p=0.01  p=0.01  p>0.999  p=0.03  F(2,19)=5.3, p>0.0001  p=0.0001  p<0.0001  p=0.9  X^2^=12.3, p=0.0007  p=0.004  p=0.013  p>0.999  X^2^=11;4, p=0.014  p=0.0029  p=0.005  p>0.999 | 8, 11, 15  8, 11, 15  8, 11, 15  8, 11  8, 15  11, 15  8, 11, 15  8, 11  8, 15  11, 15  8, 11, 15  8, 11  8, 15  11, 15  6, 9, 8  6, 9  6, 8  9, 8  6, 9, 8  6, 9  6, 8  9, 8  6, 9, 8  6, 9  6, 8  9, 8  6, 9, 8  6, 9  6, 8  9, 8 |

**Table S7: Statistical differences among the samples illustrated in Fig. 7.** Peach highlighting indicates cases when the difference is statistically significant at 0.05 level (*P*<0.05).

| Data Reference | Distribution | Type of test | Power | n |
| --- | --- | --- | --- | --- |
| **Fig. 7A**  WT age dependence  Mecp2+/- age dependence  Mecp2+/-;ob/+ age dependence  P20: WT, Mecp2+/-, Mecp2+/-;ob/+  P30: WT, Mecp2+/-, Mecp2+/-;ob/+  P40: WT, Mecp2+/-, Mecp2+/-;ob/+  WT vs Mecp2+/-  WT vs Mecp2+/-;ob/+  Mecp2+/- vs Mecp2+/-;ob/+  P50: WT, Mecp2+/-, Mecp2+/-;ob/+  WT vs Mecp2+/-  WT vs Mecp2+/-;ob/+  Mecp2+/- vs Mecp2+/-;ob/+  P60: WT, Mecp2+/-, Mecp2+/-;ob/+  WT vs Mecp2+/-  WT vs Mecp2+/-;ob/+  Mecp2+/- vs Mecp2+/-;ob/+  P100: WT, Mecp2+/-, Mecp2+/-;ob/+  WT vs Mecp2+/-  WT vs Mecp2+/-;ob/+  Mecp2+/- vs Mecp2+/-;ob/+  P200: WT, Mecp2+/-, Mecp2+/-;ob/+  WT vs Mecp2+/-  WT vs Mecp2+/-;ob/+  Mecp2+/- vs Mecp2+/-;ob/+ | Normal  Normal  Normal  Normal  Normal  Normal  Normal  Normal  Normal  Normal | ANOVA  ANOVA  ANOVA  ANOVA  ANOVA  ANOVA  Post-hoc tukey  Post-hoc tukey  Post-hoc tukey  ANOVA  Post-hoc tukey  Post-hoc tukey  Post-hoc tukey  ANOVA  Post-hoc tukey  Post-hoc tukey  Post-hoc tukey  ANOVA  Post-hoc tukey  Post-hoc tukey  Post-hoc tukey  ANOVA  Post-hoc tukey  Post-hoc tukey  Post-hoc tukey | F(6,44)=4.85, p<0.0001  F(6,59)=4.15, p<0.0001  F(6,37)=10.8, p<0.0001  F(2,14)=0.13, =0.28  F(2,18)=1.14, =0.33  F(2,19)=0.97, p=0.0018  p=0.0019  p=0.26  p=0.018  F(2,12)=2.05, =0.0012  p=0.0015  p=0.68  p=0.0064  F(2,12)=2.09, p=0.07  p=0.06  p=0.39  p=0.42  F(2,32)=50.3, p=0.01  p=0.01  p=0.69  p=0.24  F(2,32)=3.3, p<0.0001  p<0.0001  p=0.0086  p=0.34 | 5, 8, 7, 5, 4, 13, 9  5, 9, 8, 5, 6, 15, 17  6, 4, 7, 5, 6, 7, 9  5, 5, 6  8, 9, 4  7, 8, 7  7, 8  7, 7  8, 7  5, 5, 5  5, 5  5, 5  5, 5  4, 6, 6  4, 6  4, 6  6, 6  13, 15, 7  13, 15  13, 7  15, 7  9, 17, 9  9, 17  9, 9  17, 9 |
| **Fig. 7B**  P40-50: WT, Mecp2+/-, Mecp2+/-;ob/+  WT vs Mecp2+/-  WT vs Mecp2+/-;ob/+  Mecp2+/- vs Mecp2+/-;ob/+  P100-200: WT, Mecp2+/-, Mecp2+/-;ob/+  WT vs Mecp2+/-  WT vs Mecp2+/-;ob/+  Mecp2+/- vs Mecp2+/-;ob/+ | Normal  Normal | ANOVA  Post-hoc tukey  Post-hoc tukey  Post-hoc tukey  ANOVA  Post-hoc tukey  Post-hoc tukey  Post-hoc tukey | F(2,32)=3.3, p=0.055  p>0.999  p=0.025  p=0.06  F(2,62)=2.19, p=0.0004  p=0.0011  p=0.0042  p=0.97 | 14, 18, 15  14, 18  14, 15  18, 15  25, 22, 18  25, 22  25, 18  22, 18 |
| **Fig. 7C**  P40-50: WT, Mecp2+/-, Mecp2+/-;ob/+  WT vs Mecp2+/-  WT vs Mecp2+/-;ob/+  Mecp2+/- vs Mecp2+/-;ob/+  P100-200: WT, Mecp2+/-, Mecp2+/-;ob/+  WT vs Mecp2+/-  WT vs Mecp2+/-;ob/+  Mecp2+/- vs Mecp2+/-;ob/+ | Normal  Normal | ANOVA  Post-hoc tukey  Post-hoc tukey  Post-hoc tukey  ANOVA  Post-hoc tukey  Post-hoc tukey  Post-hoc tukey | F(2,16)=1.6, p=0.006  p=0.006  p=0.48  p=0.04  F(2,18)=2.47, p=0.02  p=0.022  p=0.042  p=0.94 | 5, 7, 7  5, 7  5, 7  7, 7  5, 8, 8  5, 8  5, 8  8, 8 |
| **Fig. 7D**  P40-50: WT, Mecp2+/-, Mecp2+/-;ob/+  WT vs Mecp2+/-  WT vs Mecp2+/-;ob/+  Mecp2+/- vs Mecp2+/-;ob/+  P100-200: WT, Mecp2+/-, Mecp2+/-;ob/+ | Normal  Normal | ANOVA  Post-hoc tukey  Post-hoc tukey  Post-hoc tukey  ANOVA | F(2,40)=0.11, p=0.005  p=0.033  p=0.71  p=0.0057  F(2,64)=4.12, p=0.13 | 16, 12, 15  16, 15  16, 15  12, 15  24, 23, 20 |
| **Fig. 7E**  P40: WT, Mecp2+/-, Mecp2+/-;ob/+  WT vs Mecp2+/-  WT vs Mecp2+/-;ob/+  Mecp2+/- vs Mecp2+/-;ob/+  P60: WT, Mecp2+/-, Mecp2+/-;ob/+  WT vs Mecp2+/-  WT vs Mecp2+/-;ob/+  Mecp2+/- vs Mecp2+/-;ob/+  P60: WT, Mecp2+/-, Mecp2+/-;ob/+  WT vs Mecp2+/-  WT vs Mecp2+/-;ob/+  Mecp2+/- vs Mecp2+/-;ob/+  Mecp2+/- : P40 vs P60  Mecp2+/-;ob/+: P40 vs P60 | Normal  Normal  Normal  Non-normal  Non-normal | ANOVA  Post-hoc tukey  Post-hoc tukey  Post-hoc tukey  ANOVA  Post-hoc tukey  Post-hoc tukey  Post-hoc tukey  ANOVA  Post-hoc tukey  Post-hoc tukey  Post-hoc tukey  Wilcoxon matched-pairs signed rank test  Wilcoxon matched-pairs signed rank test | F(2,19)=3.19, p=0.98,  p=0.41  p=0.08  p=0.62  F(2,19)=8.05,p=0.0008  p=0.006  p=0.04  p=0.15  F(2,12)=2.41,p<0.0001  p<0.0001  p<0.0001  p=0.82  W=-28, p=0.015  W=-12, p=0.37 | 7, 7, 7  7, 7, 7  5, 5, 5  7  7 |
| **Fig. 7F**  P60: WT, Mecp2+/-, Mecp2+/-;ob/+  Spontaneous activity  Tremor  WT vs Mecp2-/y  WT vs Mecp2y/-;ob/+  Mecp2-/y vs Mecp2y/-;ob/+  Limb grasp  WT vs Mecp2-/y  WT vs Mecp2y/-;ob/+  Mecp2-/y vs Mecp2y/-;ob/+  P150: WT, Mecp2+/-, Mecp2+/-;ob/+  Spontaneous activity  WT vs Mecp2-/y  WT vs Mecp2y/-;ob/+  Mecp2-/y vs Mecp2y/-;ob/+  Tremor  WT vs Mecp2-/y  WT vs Mecp2y/-;ob/+  Mecp2-/y vs Mecp2y/-;ob/+  Limb grasp  WT vs Mecp2-/y  WT vs Mecp2y/-;ob/+  Mecp2-/y vs Mecp2y/-;ob/+ | Non-Normal  Non-normal  Non-normal  Non-normal  Non-normal  Non-normal | Kruskal-Wallis test  Kruskal-Wallis test  Post-hoc Dunn’s  Post-hoc Dunn’s  Post-hoc Dunn’s  Kruskal-Wallis test  Post-hoc Dunn’s  Post-hoc Dunn’s  Post-hoc Dunn’s  Kruskal-Wallis test  Post-hoc Dunn’s  Post-hoc Dunn’s  Post-hoc Dunn’s  Kruskal-Wallis test  Post-hoc Dunn’s  Post-hoc Dunn’s  Post-hoc Dunn’s  Kruskal-Wallis test  Post-hoc Dunn’s  Post-hoc Dunn’s  Post-hoc Dunn’s | X^2^=0.13, p=0.25  X^2^=15.19, p<0.001  p=0.0004  p=0.095  p=0.019  X^2^=9.17, p=0.006  p=0.018  p=0.042  p>0.999  X^2^=10.45, p=0.0006  p=0.041  p=0.0069  p>0.999  X^2^=13.6, p<0.0001  p=0.003  p=0.01  p>0.999  X^2^=13.7, p<0.0001  p=0.002  p=0.01  p>0.999 | 7, 7, 7  5,5,5, |
